# Sodium selenate as a therapeutic for tauopathies: A hypothesis paper

**DOI:** 10.3389/fnagi.2022.915460

**Published:** 2022-08-05

**Authors:** Roxane Dilcher, Charles B. Malpas, Mark Walterfang, Dennis Velakoulis, Terence J. O’Brien, Lucy Vivash

**Affiliations:** ^1^Department of Neurosciences, Central Clinical School, Monash University, Melbourne, VIC, Australia; ^2^Department of Medicine and Radiology, The University of Melbourne, Melbourne, VIC, Australia; ^3^Department of Neurology, Royal Melbourne Hospital, Melbourne, VIC, Australia; ^4^Clinical Outcomes Research Unit (CORe), Department of Medicine, Royal Melbourne Hospital, The University of Melbourne, Melbourne, VIC, Australia; ^5^Neuropsychiatry Unit, Royal Melbourne Hospital, Melbourne, VIC, Australia; ^6^Department of Psychiatry and Melbourne Neuropsychiatry Center, University of Melbourne, Melbourne, VIC, Australia; ^7^Department of Neurology, Alfred Hospital, Melbourne, VIC, Australia

**Keywords:** FTLD, bvFTD, tau, sodium selenate, biomarkers, cognition

## Abstract

In a large proportion of individuals with fronto-temporal lobar degeneration (FTLD), the underlying pathology is associated with the misfolding and aggregation of the microtubule associated protein tau (FTLD-tau). With disease progression, widespread protein accumulation throughout cortical and subcortical brain regions may be responsible for neurodegeneration. One of the syndromes of FTLD is the behavioral variant of frontotemporal dementia (bvFTD), in which the underlying pathology is heterogenous, with half of the cases being related to FTLD-tau. Currently, there are no approved disease-modifying treatments for FTLD-tau, therefore representing a major unmet therapeutic need. These descriptive, preliminary findings of the phase 1 open-label trial provide data to support the potential of sodium selenate to halt the cognitive and behavioral decline, as well as to reduce tau levels in a small group of participants with bvFTD (*N* = 11). All participants were treated with sodium selenate over a period of 52 weeks. Cognition was assessed with the Neuropsychiatry Unit Cognitive Assessment Tool (NUCOG, total scores), social cognition with the Revised Self-Monitoring Scale (RSMS, total scores), behavior with the Cambridge Behavioral Inventory (CBI), and carer burden with the Caregiver Buden Scale (CBS). Fluid biomarker measures include cerebrospinal fluid of total tau (t-tau), phosphorylated tau (p-tau_181_), NfL, p-tau_181_/t-tau, t-tau/Aβ_1–42_, and p-tau_181_/Aβ_1–42_ levels. After treatment at follow-up, cognition and behavior showed further negative change (based on a reliable change criterion cut-off of annual NUCOG decline) in the “progressors,” but not in the “non-progressors.” “Non-progressors” also showed elevated baseline CSF tau levels and no increase after treatment, indicating underlying tau pathology and a positive response to sodium selenate treatment. Significant changes in MRI were not observed. The findings provide useful information for future clinical trials to systematically assess the disease-modifying treatment effects of sodium selenate in randomized controlled designs for bvFTD and FTLD-tau pathologies.

## Introduction

Fronto-temporal lobar degeneration (FTLD) is an umbrella term for the underlying pathology of a range of neurodegenerative disorders, including progressive supranuclear palsy (PSP), corticobasal syndrome (CBS), and frontotemporal dementia (FTD). FTD can be further classified into the more common behavioral variant FTD (bvFTD), which is marked by changes in behavior, emotion, personality, and executive functions, as well as non-fluent/agrammatic primary progressive aphasia (nfvPPA), and semantic variant primary progressive aphasia (svPPA), with the latter two being classified according to different patterns of language impairments (Neary et al., 1998). The different entities of FTLD are defined by their initial clinical presentation with differing cognitive, behavioral, and neuropsychiatric profiles (Murley et al., 2020). With increasing neurodegeneration, clinical features and common atrophy patterns in mainly frontal and/or temporal lobes overlap between disorders, leading to increased difficulties in the detection of distinct markers and accurate and timely diagnosis, which can cause severe personal, social and financial distress to patients and their families. Although longitudinal biomarker assessment exists for PSP (Whitwell et al., 2019), studies that track markers of disease progression in bvFTD are currently lacking, as are studies that investigate differences between pathological causes. There are currently no approved disease-modifying treatments for bvFTD, representing a major unmet need in the treatment of this rare condition.

## Pathology of FTLD

Various underlying pathogenic proteins and processes are involved in disease propagation. Abnormal protein accumulation of the microtubule associated protein tau (FTLD-tau) and TDP-43 (FTLD-TDP) in glia and neurons throughout cortical and subcortical brain regions are the main causes of neurodegeneration and neuropsychological decline in FTLDs (Kovacs, 2018). These two proteins explain approximately 90% of FTLD cases (Snowden et al., 2007). Most cases are sporadic, but some can be caused by rare genetic mutations. For instance, FTLD-tau is caused by the microtubule-associated tau gene, *MAPT*, while FTLD-TDP is associated with either *C9orf72* or *GRN* mutations (Irwin et al., 2015). In FTDL-TDP pathologies, the nuclear protein TDP-43 aggregates to the cytoplasm where it forms cytoplasmic inclusions (Irwin et al., 2015). It was suggested that in FTLD-tau, abnormal hyperphosphorylated tau aggregates detach from microtubules by propagating transcellularly and extracellular in the cerebrospinal fluid (CSF) and thereby spreading tau pathology into different brain areas as the disease progresses (Brunello et al., 2020). However, the exact mechanisms of tau propagation are largely unclear. Deposited tau is characterized by 3 repeat tau isoforms (3R) in bvFTD, while 4R can be present in PSP and CBS (Kovacs, 2018).

Progressive supranuclear palsy, CBS, and svPPA are primarily associated with FTLD-tau, but in bvFTD, the underlying pathology is heterogenous, and therefore, the relationship between clinical presentation and pathology is poorly understood. To date, cognitive impairments, including memory, executive functioning, visuo-construction, language, and behavioral changes have been associated with tau pathology or *MAPT* mutation (Hu et al., 2007; Grossman et al., 2008; Mendez et al., 2013; Kamath et al., 2019; Poos et al., 2020; Benussi et al., 2021), while social cognition impairments were found in patients with *C9orf72* (Russell et al., 2020; Franklin et al., 2021).

## Current biomarkers of bvFTD

The urgent need for disease-modifying treatment of bvFTD has increased the interest in sensitive non-invasive markers of disease and disease progression. Despite pathological confirmation of tau or TDP-43, fluid biomarkers have yet not been validated as robust disease-specific markers. High levels of neurofilament light chain (NfL) in CSF and blood show high sensitivity for identifying patients with neurodegenerative diseases compared to healthy controls, but this is neither specific to bvFTD nor to tau or TDP-43 in general (Vijverberg et al., 2017; Karantali et al., 2020).

Phosphorylated tau (p-tau_181_) levels in CSF correlate with cognition and cognitive decline in cases with FTLD-tau (Koedam et al., 2013; Rojas et al., 2018). In bvFTD, both total tau (t-tau) and p-tau_181_ levels are lower than in Alzheimer’s disease (AD) (Bian et al., 2008), and there is evidence that the ratio of p-tau_181_ to t-tau in CSF is lower in FTLD-TDP than in FTLD-tau (Hu et al., 2013; Borroni et al., 2015).

Plasma p-tau_217_ or p-tau_181_ has the potential to discriminate AD from FTLD syndromes (Thijssen et al., 2021), but whether p-tau or t-tau levels are low or high among different FTLD subtypes remains unclear. Although there exist reliable fluid biomarkers for AD (Bălaşa et al., 2020), the differential diagnosis for FTLD subtypes based on CSF or blood biomarkers of tau or TDP-43 remains limited and inconclusive (Vijverberg et al., 2017).

In recent years, tau-PET tracers have been increasingly developed for selectively targeting tau pathology *in vivo*. As a result, tau PET imaging showed superior diagnostic validity to CSF tau biomarkers in AD (Mattsson et al., 2018) and correlated with CSF tau levels and cognitive deficits (Saint-Aubert et al., 2016; Bejanin et al., 2017; Chiotis et al., 2018; La Joie et al., 2018). Recently developed second-generation tracers, such as PI-2620, show high sensitivity for detecting tau accumulation in AD and FTLD-tau cases, such as 4R-related PSP and CBS (Brendel et al., 2020; Palleis et al., 2021). For bvFTD, the studies of newly developed tau tracers that enable reduced off-binding potential are currently limited. The difficulty in finding bvFTD-specific biomarkers may arise from their heterogenous pathologies. Current research gaps exist because PET imaging is expensive, not easily available, and no tau-tracer is yet approved for clinical use.

## Sodium selenate as a disease-modifying therapy – animal studies

Due to a combination of a lack of diagnostic and prognostic biomarkers, uncertain therapeutic targets and disease heterogeneity, there have been no successful international trials of novel drug treatments to treat the underlying pathology in bvFTD. Current treatment strategies aim at managing neuropsychiatric symptoms (see review, Le and Finger, 2021). A multisite, phase 2 double-blind, randomized, placebo-controlled trial is currently underway (Vivash et al., 2020). A large body of pre-clinical and emerging clinical data has provided evidence for sodium selenate, which is an oxidized, less toxic salt of selenium, as a promising disease-modifying treatment strategy for hyperphosphorylated tau-based diseases (Corcoran et al., 2010; van Eersel et al., 2010; Zheng et al., 2014; Shultz et al., 2015; Brady et al., 2016; Malpas et al., 2016; Tan et al., 2016). Sodium selenate activates the protein phosphatase 2a (PP2A) enzyme, the major tau serine/threonine phosphatase present in the brain (Goedert et al., 1995; Gong et al., 2000), which reduces levels of hyperphosphorylated tau. For example, previous work on transgenic AD models demonstrated that high-dose sodium selenate significantly impacts tau aggregation by upregulating PP2A/PR55, thereby dephosphorylating tau and reducing p-tau and t-tau levels in the hippocampus and amygdala (Corcoran et al., 2010), and reversed spatial learning, memory, and motor deficits (Corcoran et al., 2010; van Eersel et al., 2010). Similar beneficial effects have been demonstrated in rodent models of tau-related conditions in traumatic brain injury and epilepsy (Jones et al., 2012; Shultz et al., 2015; Brady et al., 2016; Tan et al., 2016). For instance, previous work of our group showed that continuous sodium selenate treatment increased PP2A/PR55, reduced p-tau, t-tau, p-tau/t-tau, and brain damage, and improved behavioral, motor, and cognitive outcomes in rat models with induced traumatic brain injury (Shultz et al., 2015; Brady et al., 2016; Tan et al., 2016). Moreover, sodium selenate treatment was effective in inhibiting seizures in amygdala kindled rats (Jones et al., 2012).

## Evidence from previous clinical studies

Sodium selenate represents a potential disease-modifying treatment for hyperphosphorylated tau-based diseases in humans (Malpas et al., 2016; Cardoso et al., 2019; Vivash et al., 2021, 2022). In patients with mild to moderate AD, treatment with sodium selenate was safe and well-tolerated, and treated patients showed less deterioration on diffusion MR. However, no evidence for differences in cognition, CSF, or MRI measures was found between the treatment and control groups, potentially due to the short treatment period (Malpas et al., 2016). Follow-up studies on patients from this trial found that higher selenium levels (in the CSF and blood) were associated with no change in cognitive measures, whereas those with cognitive decline did not have the same increase in fluid selenium levels (Cardoso et al., 2019). Moreover, an open-label extension study showed sodium selenate treatment to be safe and well-tolerated when taken long term (10–22 months), with lesser cognitive decline than would be expected in the natural history of AD (Vivash et al., 2021). Given the promising safety profile and mechanism of action, sodium selenate represents an effective treatment for FTLD-tau. A recent open-label phase 1b study by our group reported sodium selenate to be safe and well-tolerated in patients with bvFTD with a divergence of brain atrophy rates and cognitive change within the patient cohort (Vivash et al., 2022). In the previous study, results were reported based on predefined protocol analysis and by subdividing patient groups based on annual whole brain volume changes. Using the same sample, we herein add further descriptive exploratory findings that support our hypothesis that sodium selenate may be a disease-modifying treatment of FTLD-tau.

## Sodium selenate as a treatment of bvFTD is an ongoing investigation

The following descriptive analyses report the effect of 12 months of treatment with sodium selenate on a group of patients diagnosed with bvFTD (*n* = 11). The study design and intervention, patient demographics, and safety results are reported elsewhere (Vivash et al., 2022). The majority of patients (55%) did not show a substantial decline in a range of cognitive measures and biomarkers. However, disease progression was evident in a minority of patients (45%). We, therefore, hypothesized that the differences in progression rate were due to different underlying pathology, with only one group of patients responding to the anti-tau treatment. To investigate this, participants were stratified according to their annual cognitive change. A change in total NUCOG scores in bvFTD has been demonstrated as a reliable marker of cognitive decline in dementia (Walia et al., 2022). The standardized method of Jacobson et al. (1984) allows classifying patients according to different treatment outcomes by using a reliable change criterion. The formula was adapted to our data to calculate the annual NUCOG change. We included the total NUCOG standard deviation of the original control sample, which is ± 4.88 (Walterfang et al., 2006), and selected a reliability index of 0.88. The cut-off value for the group stratification was then calculated with the following formula at the 95% level:


$$1.96\times \mathrm{SD1}\times \sqrt{2}\times \sqrt{1-\mathrm{rel}}=\mathrm{Reliable}\,\mathrm{change}\,\mathrm{criterion}$$

$$1.96\times 4.88\times \sqrt{2}\times \sqrt{1-0.88}=\pm 4.69$$

The group with an absolute NUCOG total change lesser than the calculated reliable change criterion of −4.69 were classified as “non-progressors” to sodium selenate treatment. The “progressors” showed a reliable performance decrease of more than −4.69. This resulted in *n* = 6 “non-progressors” and *n* = 5 “progressors” in line with the reported distribution of underlying tau and TDP-43 pathologies [approx. 50:50 (5)]. Moreover, the patients with MAPT and C9Orf72 mutations were classified into the “correct” group as per our hypothesis. Baseline scores and biomarker levels were taken into account to test if different initial disease stages explain the different progression rates.

### Neuropsychological findings

Given the potential beneficial effect of sodium selenate on increasing PP2A activity and reducing hyperphosphorylated tau levels in tau pathologies, as well as the consecutive benefit on cognition and behavior, it is hypothesized that participants who respond to treatment (non-progressors) experience less disease burden than progressors over time, as reflected by less negative changes in cognition and behavior. The tau-unspecific marker of social cognitive decline is expected to decline in both groups in a similar fashion.

**FIGURE 1:**
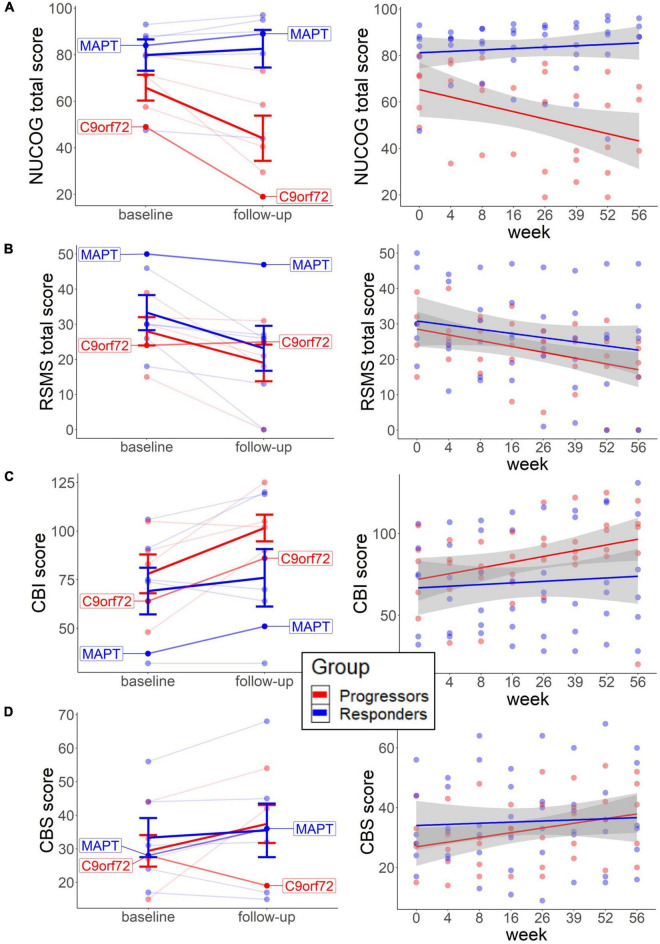
Baseline scores and long-term neuropsychological change in each group. The left graphs represent the change from baseline to follow-up on the individual level for cognition **(A)**, social cognition **(B)**, behavior **(C)**, and carer burden **(D)**. Thick lines reflect group means. The right graphs **(A–D)** represent changes across multiple weeks from baseline to follow-up, with each line representing each group. Both patients with the identified mutation are highlighted. NUCOG, Neuropsychiatry Unit Cognitive Assessment Tool (total scores); RSMS, Revised Self-Monitoring Scale (total scores); CBI, Cambridge Behavioral Inventory; CBS, Caregiver Buden Scale.

**FIGURE 2:**
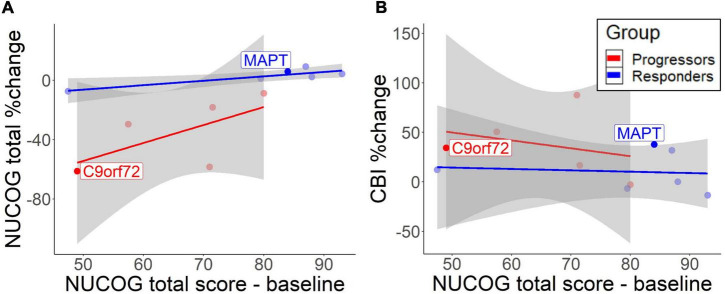
Correlation between baseline total NUCOG scores and cognitive decrease **(A)** and behavioral change **(B)**. The lines represent different groups. Results are based on bootstrapped Pearson correlations. Both patients with the identified mutation are highlighted.

The neuropsychological findings (Figures 1, 2) point to the beneficial treatment effect of sodium selenate on cognition and behavior in participants who responded to tau-treatment and who may therefore present an underlying tau-pathology. At baseline, independent *t*-test showed slightly elevated cognition (*M* = 79.8, *SD* = 16.5, and *p* = 0.121; Mann–Whitney *U*) and social cognition scores (*M* = 33.3, *SD* = 12.2, and *p* = 0.645) and fewer behavioral impairments (*M* = 69.2, *SD* = 29.4, and *p* = 0.784) in the non-progressors, than in the progressors (NUCOG: *M* = 65.8, *SD* = 12.4; RSMS: *M* = 28.0, *SD* = 9.0; CBI: *M* = 78.0, *SD* = 22.3) (Figures 1A–C). No difference was observed for carer burden scores (Figure 1D).

Dependent *t*-test revealed that the NUCOG scores decreased in the progressors (*M* = −35.2, *SD* = 23.7, and *p* = 0.059; Wilcoxon signed-rank), while a slight increase was observed in the non-progressors (*M* = 2.6, *SD* = 6.6, and *p* = 0.142) from baseline to follow-up (Figure 1A, left). Similarly, descriptive analysis of the time effects across multiple time points showed a trend of decrease in NUCOG scores in the progressors, and no change in the non-progressors over weeks 0, 4, 8, 16, 26, 36, 52, and 56 (Figure 1A, right). We could tentatively assume that cognitive decline, as measured by total NUCOG scores, and changes in behavior reflect tau-related processes. This is further supported by the trajectories of the two patients with genetic mutations, as the patient with the *MAPT* mutation showed a higher baseline and long-term increase of NUCOG, compared to the patient with the *C9orf72* expansion.

In contrast, RSMS scores decreased significantly in the non-progressors (*M* = −33.4, *SD* = 35.4, and *p* = 0.036) and slightly in the progressors (*M* = −36.6, *SD* = 42.3, and *p* = 0.136.) over time (Figure 1B, left) and also tended to decrease in both groups in a similar fashion over multiple time points (Figure 1B, right). Previous findings showed more social cognitive impairments in patients with early presymptomatic *C9orf72* compared to *MAPT* (Russell et al., 2020; Franklin et al., 2021), highlighting that social cognitive impairments may not be a typical marker of underlying tau pathology. This may explain why, in our study, sodium selenate treatment does not protect against social cognitive decline in the non-progressors.

Scores in the CBI and CBS slightly increased in the progressors over time (CBI change: *M* = 37.3, *SD* = 34.4, and *p* = 0.104; CBS change: *M* = 42.6, *SD* = 80.7, and *p* = 0.223), which was less pronounced in the non-progressors (CBI change: *M* = 10.3, *SD* = 20.9, and *p* = 0.281; CBS change: *M* = 2.4, *SD* = 21.2, and *p* = 0.528) (Figures 1C,D, left) with similar findings across multiple time-points (Figures 1C, 2D, right).

A descriptive analysis of correlations suggested that lower total NUCOG baseline predicted cognitive decline over time in the progressors [*r* = 0.63, 95% CI (−0.10, 1.40)] and to a lesser extent in non-progressors [*r* = 0.87, 95% CI (0.23, 1.89)] (Figure 2A). However, the high standard deviation in the progressors does not allow reliable significance testing. CBI measures showed that low cognition scores predicted behavioral impairment increase in the progressors [*r* = −0.89, 95% CI (−1.47, −0.42)], but no correlation was found in non-progressors [*r* = −0.06, 95% CI (−1.05, 0.82)] (Figure 2B). Therefore, higher baseline cognition protects from the cognitive and behavioral decline in the progressors.

The different pattern of neuropsychological decline in both groups is supported by a large body of research, showing more cognitive impairment (memory, executive functioning, visuo-construction, and language) and more behavioral/personality changes in tau pathology or *MAPT* mutation carriers compared to tau-negative cases or *GRN*/*C9orf72* carriers (Hu et al., 2007; Grossman et al., 2008; Mendez et al., 2013; Kamath et al., 2019; Poos et al., 2020; Benussi et al., 2021). Conflicting results showed greater executive dysfunction in *GRN* mutation carriers (Poos et al., 2020) and confrontation naming difficulties in tau-negative FTLD (Grossman et al., 2008).

### Fluid biomarker findings

Given the known effect of sodium selenate on PP2A and downstream impacts on tau and p-tau levels demonstrated in animal models of disease and the known accumulation of tau and p-tau in patients with FTLD-tau, it is hypothesized that CSF tau-related markers (t-tau, p-tau, and p/t-tau ratio) are expected to be reduced or halted from a further increase in the non-progressors as a response to treatment and increased in the progressors. CSF changes are related to different rates of annual neuropsychological decline between groups.

**FIGURE 3:**
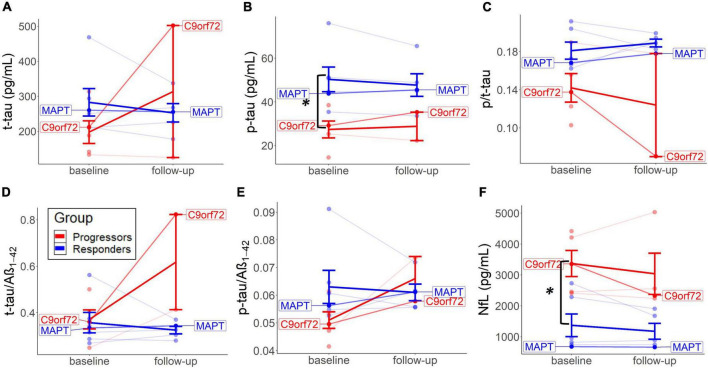
Baseline and long-term CSF change in each group. Changes from baseline to follow-up on the individual level are represented for t-tau **(A)**, p-tau **(B)**, p/t-tau **(C)**, t-tau/Aβ_1–42_ **(D)**, p-tau/Aβ_1–42_ **(E)**, and NfL levels **(F)**. Thick lines reflect group means. Both patients with the identified mutation are highlighted.

Baseline CSF tau-levels were generally higher in the non-progressors, with independent *t*-tests revealing significant higher p-tau levels (*M* = 50.3, *SD* = 13.8, and *p* = 0.014; Mann–Whitney *U*); and slightly higher t-tau (*M* = 282.9, *SD* = 96.2, and *p* = 0.121), p-tau/t-tau (*M* = 0.18, *SD* = 0.02, and *p* = 0.055), and p-tau/Aβ_1–42_ ratios (*M* = 0.06, *SD* = 0.01, and *p* = 0.083) compared to the progressors (p-tau: *M* = 0.18, *SD* = 0.02; t-tau: *M* = 27.4, *SD* = 8.6; t-tau: *M* = 197.7, *SD* = 72.5; p/t-tau: *M* = 0.14, *SD* = 0.03; p-tau/Aβ_1–42_: *M* = 0.05, *SD* = 0.01) (Figures 3A–C,E). On the contrary, baseline CSF NfL values were significantly lower in the non-progressors (*M* = 1371.4, *SD* = 896.3, and *p* = 0.022), compared to the progressors (*M* = 3370.3 and *SD* = 942.8) (Figure 3F).

Fluid biomarker levels changed to a different extent per group after treatment. Descriptive analyses suggested that p/t-tau ratio levels decreased in both groups but did more so in the progressors. No significant testing could be performed because of missing values. The non-progressors tended to experience a decrease in t-tau and p-tau from baseline to follow-up, compared to an increase of these values in the progressors (Figures 3A,B). T-tau/Aβ_1–42_ and p-tau/Aβ_1–42_ ratios increased in the progressors but did not show a particular change over time in the non-progressors (Figures 3D,E). P/t-tau and NfL levels did not change in any particular direction, but both groups showed a similar trend of decrease in NfL over time (Figures 3C,F). Interestingly, CSF values in the two mutation carriers (*MAPT* and *C9orf72*) correspond to the group means of the non-progressors and progressors, respectively (Figure 3).

There is growing evidence for distinct protein biomarker profiles to aid in the differentiation of FTLD-tau and FTLD-TDP. The higher baseline tau-levels (Figure 3) could indicate an underlying tau-pathology. Interestingly, lower baseline p/t-tau levels in the progressors could suggest that this group is associated with underlying TDP-43 (Hu et al., 2013; Borroni et al., 2015). As a result, it is plausible that higher baseline NfL levels in the progressors similarly reflect a marker of FTLD-TDP. Previous studies found elevated NfL in several neurodegenerative diseases, mainly associated with amyotrophic lateral sclerosis (ALS), FTD, TDP-43 (Olsson et al., 2019), and *GRN* and not with *C9or72* or *MAPT* (Rojas et al., 2021). Furthermore, our previous validation study found similar results to indicate low p/tau ratio and high NfL as a marker of underlying FTLD-TDP (Vivash et al., 2022).

**FIGURE 4:**
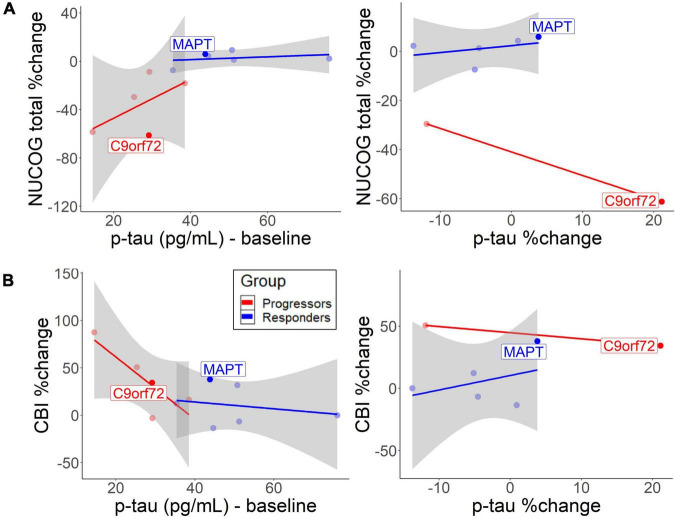
Correlation between baseline/change CSF p-tau levels and **(A)** cognitive decrease and **(B)** behavioral change. The lines represent different groups. The left graphs represent p-tau baseline levels and the right graphs represent annual p-tau changes. Both patients with the identified mutation are highlighted.

Correlation analysis between baseline CSF biomarkers and neuropsychological tests were performed with p-tau values, as they were the best in discriminating the non-progressors from the progressors. Similar results were found for t-tau (results not shown). Cognition, behavior, and carer burden changed as a function of baseline CSF levels. The descriptive analysis suggested that lower baseline p-tau levels in the progressors slightly predicted cognitive decline [*r* = 0.59, 95% CI (−0.14, 1.42)] (Figure 4A, left) and behavioral decline [*r* = −0.83, 95% CI (−1.46, −0.26)] (Figure 4B, left). In the non-progressors, the group with the higher baseline p-tau levels, no such correlation was seen for cognitive change [*r* = 0.28, 95% CI (−0.72, 1.40)] or behavior [*r* = −0.24, 95% CI (−0.96, 0.43)]. The responder group, who only experienced p-tau decrease after treatment, did not exhibit a cognitive [*r* = 0.37, 95% CI (−0.70, 1.12)] or behavioral decline [*r* = 0.39, 95% CI (−0.73, 1.84)] as a function of p-tau change (Figure 4B, right). To a small extent, an increase in p-tau predicted cognitive decline in the progressors (Figure 4A, right), but no correlation was observed for behavioral change. No bootstrap correlation between p-tau change and neuropsychological change could be performed for the progressors, due to missing values.

While baseline p-tau levels, as well as annual increases in p-tau and t-tau predict disease burden in the progressors, non-progressors did not exhibit negative cognitive and behavioral changes (Figure 4). The findings support previous findings that CSF p-tau correlates with cognition and cognitive decline in FTLD-tau (Koedam et al., 2013; Rojas et al., 2018). Therefore, the higher baseline and more pronounced decline rate of p-tau and t-tau in the non-progressors could indicate an underlying tau-pathology. Furthermore, the findings suggest that sodium selenate protected the non-progressors from a further increase in t-tau or p-tau levels over time. The results are similar to a previous study, which found no longitudinal increase of p-tau and t-tau in those treated with sodium selenate in an AD cohort (Malpas et al., 2016). However, the results show that sodium selenate treatment does not have a protective function on the decline in social cognition, suggesting that social cognition may be a tau-unspecific marker of disease progression. Lower baseline p/t-tau ratios and the rate of decline in the progressors suggest that this group may have underlying TDP-43 (Hu et al., 2013; Borroni et al., 2015). NfL slightly tended to decrease after sodium selenate treatment in a similar fashion in both groups, which could reflect a general marker of neurodegeneration, not specific to tau pathology. It has been reported that higher NfL levels are related to higher cognitive and behavioral impairments and a more rapid decline in neurodegenerative diseases, thus serving as a diagnostic marker of neurodegeneration more generally (Olsson et al., 2019; Rojas et al., 2021; Walia et al., 2022). Higher levels have also been specifically related to TDP-43 pathology (Olsson et al., 2019). However, the relationship between NfL levels and active vs. total neurodegeneration and how NfL levels relate to disease progression and/or spread remains to be determined.

It should be noted, that rather than reflecting different underlying pathologies, lower baseline NfL in the non-progressors could point to this group being recruited at an early disease stage, compared to the other group. Lower levels of NfL, and therefore of neurodegeneration, could also explain the overall better cognitive baseline performance (and higher brain volumes) in this group and suggest that patients in early stages benefit more from treatment over time than patients with the severely progressed disease. Against this is the finding that different levels of baseline p-tau, t-tau, and p/t-tau values, and different patterns of disease progression in both groups correspond to the typical profile of FTLD-tau. Moreover, the two identified participants with underlying *MAPT* and *C9orf72* confirm the correct group-stratification of underlying tau and TDP-43, respectively.

### Brain volume findings

Elevated atrophy in widespread brain areas is expected in progressors over time, due to increasing neurodegeneration in FTLD. Lesser atrophy is expected in those who respond to treatment. Associations between brain volume and cognitive decline will be explored.

There was an overall trend of higher baseline MRI measures in all brain areas in the non-progressors, but *t*-tests for group comparison were not significant, except for the superior frontal areas (*M* = 1.41, *SD* = 0.12, *p* = 0.022, Mann–Whitney *U*). Higher baseline volumes in all areas in the non-progressors could indicate that the disease is progressed to a lesser extent than in the progressors at entry into the trial, which would correspond to better cognitive and behavioral scores, as well as to lower baseline NfL levels (see the previous section). The positive treatment effects observed in the non-progressors could therefore be explained by an early treatment benefit. This highlights the importance of detecting tau-pathologies early to obtain maximum treatment benefit.

**FIGURE 5:**
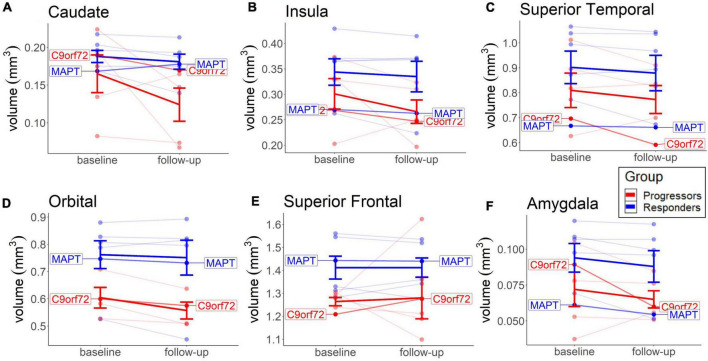
Baseline and long-term MRI brain volume change in each group. Depicted are the Caudate **(A)**, Insula **(B)**, Orbital **(C)**, and Superior Frontal region **(D)**, because of more visible group differences, compared to other brain areas. Thick lines reflect group means. Both patients with the identified mutation are highlighted.

No significant group differences or significant volume changes over time were seen in any brain area, contrary to our hypothesis. A descriptive analysis of the changes shows a slightly higher rate of decrease in the progressors in the caudate (*M* = −19.7, *SD* = 10.8), insula (*M* = −8.35, *SD* = 7.92), and orbital regions (*M* = −7.47, *SD* = 4.08), compared to the non-progressors (caudate: *M* = −4.48, *SD* = 1.88; insula: *M* = −3.24, *SD* = 0.38; orbital: *M* = −2.41, *SD* = 1.40) (Figure 5). Most of the other brain areas show a similar rate of decline in both groups (e.g., superior temporal and amygdala) or no decline in either group (e.g., superior frontal cortex) (Figure 5). The lower rate of brain volume decreases in the non-responders, compared to the progressors, in some areas (Figure 5), points to a putative beneficial effect of sodium selenate on neurodegeneration in patients with tau pathology.

The cognitive decline over time was not predicted by lower brain volume in the caudate, insula, orbital, or superior frontal cortex in any group (results not shown). The lack of associations between annual volume decline and cognitive decline in these areas suggests that these brain areas were not typically affected by TDP-43 pathology and that sodium selenate may have provided a beneficial effect on tau pathology. The positive effect of sodium selenate on preventing deterioration in axonal structural integrity was previously found in patients with AD (Malpas et al., 2016).

In both groups, cognitive decline was predicted by higher volume decrease in the amygdala [non-progressors: *r* = 0.61, 95% CI (−0.50, 2.43); progressors: *r* = 0.69, 95% CI (−0.03, 1.29)], pericalcarine [non-progressors: *r* = 0.77, 95% CI (−0.20, −2.24); progressors: *r* = 0.45, 95% CI (−0.68, 1.56)], precentral [non-progressors: *r* = 0.74, 95% CI (−0.10, 1.83); progressors: *r* = 0.77, 95% CI (0.00, 1.58)], fusiform [non-progressors: *r* = 0.89, 95% CI (−0.20, 1.87); progressors: *r* = 0.621, 95% CI (−0.20, 1.50)], and superior temporal cortex [non-progressors: *r* = 0.97, 95% CI (0.54, 1.49); progressors: *r* = 0.631, 95% CI (−0.30, 1.603)]. No specific correlation patterns were detected for behavior. The findings of similar neural correlates in both groups are consistent with studies who found these areas to be part of the typically deregulated prefrontal-paralimbic structures in bvFTD more generally and to be related to executive dysfunction and memory and social impairments (Irish et al., 2014; Kamminga et al., 2015; Dermody et al., 2016; Baez et al., 2019). No group difference and therefore no effect on tau was found. Some of these dysregulated networks could mirror the decline in social cognition in our study, which in turn was found to be unspecific to any group. For example, previous studies explained impairments in social cognition by a volume decrease in the amygdala (De Winter et al., 2016; Dermody et al., 2016; Baez et al., 2019) and impairments in social cognition were higher in patients with C9orf72, compared to MAPT (Russell et al., 2020; Franklin et al., 2021).

An overall lack of group differences in MRI could also be explained by a low treatment effect of sodium selenate on brain volume change over a period of 52 months. It is conceivable that neurodegeneration is not visible at this early time point on MRI. On the contrary, CSF values could be more reliable in detecting early markers of disease propagation, but the overall non-significant results in this study indicate that a longer time period could be needed to observe significant effects on MRI or liquid biomarkers in bvFTD. PET scanning may serve as a promising future diagnosis technique and can detect tau pathology in its early stages (Mattsson et al., 2018; Brendel et al., 2020).

## Summary of findings and future directions

The results of this phase 1 open-label study provide new data to support the ongoing investigation of sodium selenate in FTLD, demonstrating the impact on tau levels and the concomitant effects on cognition, behavior, and pathological neurodegeneration. Descriptive analyses demonstrate the potential of the treatment to halt the cognitive and behavioral decline, as well as to reduce p-tau and t-tau levels in a subset of patients with bvFTD. A decline in social cognition did not seem to be affected by sodium selenate treatment and no change was found on MRI measures. The reduced disease progression pattern and the elevated baseline tau-levels confirm that our two groups may have been correctly stratified according to the presence or absence of underlying tau and provide evidence that one subset of patients responds to tau treatment. The main limitation of the study is the small group size, which prevents a powerful quantitative analysis. Moreover, this open-label study neither includes a control group nor includes tau PET or histopathological findings that would add important information about the underlying pathology. However, the current findings can guide future trials that aim to demonstrate the disease-modifying treatment effects of sodium selenate on patients with underlying tau. A current multisite, phase 2 double-blind, randomized, placebo-controlled trial with a higher number of participants is currently underway (Vivash et al., 2020). The current findings suggest that future longitudinal studies should take baseline levels of biomarkers and cognitive performance into account, as well as different disease stages, to verify a beneficial treatment effect on patients with less severe disease stages.

## Data availability statement

Study data will be available upon reasonable academic requests subject to human ethics committee approval. Requests should be directed to the corresponding author and/or human ethics committee. Requests to access the datasets should be directed to LV, lucy.vivash@monash.edu.

## Ethics statement

The studies involving human participants were reviewed and approved by Melbourne Health Human Research Ethics Committee (local reference 2017.090). Written informed consent was obtained from participants or their legally authorized representative and their study partner.

## Author contributions

CM, MW, DV, TO’B, and LV conceived and designed research and edited and revised the manuscript. RD analyzed the data, drafted the manuscript, and prepared figures. RD, CM, TO’B, and LV interpreted results. All authors contributed to the article and approved the final manuscript.
